# Identification of (*Z*)-8-Heptadecene and *n*-Pentadecane as Electrophysiologically Active Compounds in *Ophrys insectifera* and Its *Argogorytes* Pollinator

**DOI:** 10.3390/ijms21020620

**Published:** 2020-01-17

**Authors:** Björn Bohman, Alyssa M. Weinstein, Raimondas Mozuraitis, Gavin R. Flematti, Anna-Karin Borg-Karlson

**Affiliations:** 1 School of Molecular Sciences, the University of Western Australia, Crawley, WA 6009, Australia; 2 Department of Plant Protection Biology, Swedish University of Agricultural Sciences, 23053 Alnarp, Sweden; 3 Research School of Biology, the Australian National University, Acton, ACT 2601, Australia; 4 Department of Zoology, Stockholm University, 106 91, Stockholm, Sweden; 5 Department of Chemical Engineering, Mid Sweden University, 85170 Sundsvall, Sweden

**Keywords:** *Ophrys*, sexual deception, semiochemicals, fly orchid, pollination

## Abstract

Sexually deceptive orchids typically depend on specific insect species for pollination, which are lured by sex pheromone mimicry. European *Ophrys* orchids often exploit specific species of wasps or bees with carboxylic acid derivatives. Here, we identify the specific semiochemicals present in *O. insectifera*, and in females of one of its pollinator species, *Argogorytes fargeii*. Headspace volatile samples and solvent extracts were analysed by GC-MS and semiochemicals were structurally elucidated by microderivatisation experiments and synthesis. (*Z*)-8-Heptadecene and *n*-pentadecane were confirmed as present in both *O. insectifera* and *A. fargeii* female extracts, with both compounds being found to be electrophysiologically active to pollinators. The identified semiochemicals were compared with previously identified *Ophrys* pollinator attractants, such as (*Z*)-9 and (*Z*)-12-C_27_-C_29_ alkenes in *O. sphegodes* and (*Z*)-9-octadecenal, octadecanal, ethyl linoleate and ethyl oleate in *O. speculum*, to provide further insights into the biosynthesis of semiochemicals in this genus. We propose that all these currently identified *Ophrys* semiochemicals can be formed biosynthetically from the same activated carboxylic acid precursors, after a sequence of elongation and decarbonylation reactions in *O. sphegodes* and *O. speculum*, while in *O. insectifera*, possibly by decarbonylation without preceding elongation.

## 1. Introduction

Pseudocopulation as a means of pollination was first reported over 100 years ago, in two parallel systems [1,2]. Correvon and Pouyanne made observations of European *Ophrys* orchids [1], while in Australia, *Cryptostylis* orchids were reported to use the same sexually deceptive strategy, in which insect pollinators attempt copulatory or courtship behaviour with the flower, thereby transferring pollinia [2,3]. Insect sexual attraction is induced through chemical and physical mimicry of female insects. Pollination by sexual deception is now known to be a phenomenon that has evolved independently multiple times on different continents. There are several hundred confirmed cases in the Orchidaceae, with many more likely to be discovered with future studies [4,5,6]. There are also single reports of sexual deception in the Asteraceae [7] and Iridaceae [8], indicating that this pollination strategy may be more common than is currently known.

Following the initial observations of pollination via sexual deception in *Ophrys* and *Cryptostylis* orchids, an intensive Swedish research program was launched in 1948 to investigate the chemical cues underlying this bizarre pollination strategy. *Ophrys insectifera* and some southern European *Ophrys* and their solitary bee pollinator species were the main study species [9]. In these early studies, field experiments demonstrated that floral volatiles were the key to pollinator attraction [9,10]. With the use of electroantennography (EAG), it was later shown that two species of male sphecid wasp pollinator, *Argogorytes mystaceus* and *A. fargeii*, unlike their conspecific females, responded to tentatively identified alkanes, alkenes, and terpenes in sorption headspace extracts of *O. insectifera* flowers [11]. A few years later, the first evidence of chemical mimicry of several species of *Andrena* bee pollinators by *O. fusca* and *O. lutea*, was found: aliphatic alcohols, monoterpene- and sesquiterpene alcohols, and aldehydes attracted the patrolling males to varying degrees [12,13]. 

The first identification of pollinator sexual attractants in the genus *Ophrys* did not occur until the late 1990s, with the successful structural elucidation of attractants from *O. sphegodes* [14,15]. A key to the detection and identification of the semiochemicals from this species was the use of gas chromatography coupled with electroantennogram detection (GC-EAD), which revealed a set of 14 electrophysiologically active compounds to be shared among the orchid and the female of its bee pollinator, *Andrena nigroaenea*. Before being confirmed as attractants in field bioassays, these compounds were identified by GC-MS, including microderivatisation experiments, as a series of long-chained alkanes and alkenes. Furthermore, three (*Z*)-7 alkenes were discovered to be responsible for the attraction of male *Colletes cunicularius* bees to *O. exaltata* [16]. The chemical stimuli for the sexual attraction of various *Ophrys* pollinators also include other types of structures, as shown when a mixture of hydroxy- and keto acids, together with aldehydes and esters, were identified as the attractants in *O. speculum*, which is pollinated by male *Campsoscolia ciliata* scoliid wasps [17]. 

In Australian sexually deceptive orchids, 1,3-cyclohexanediones (chiloglottones) have been identified as pollinator attractants in *Chiloglottis* [18], as have hydroxymethylpyrazines and a β-hydroxylactone (drakolide) in *Drakaea* [19,20,21,22], (methylthio)phenols, acetophenones and monoterpenes in *Caladenia* [23,24,25], and tetrahydrofuran acid derivatives in *Cryptostylis* [26].

Besides the discovery of a broad range of compounds pivotal for pollination in sexually deceptive orchids, there has also been interest in the biosynthesis of these compounds, with the aim to link biosynthesis to the evolution and speciation of orchids. Schlüter and Schiestl [27] predicted that, in *Ophrys,* the biosynthesis of alkenes would follow the biosynthetic pathway for alkanes [28], but with the addition of an extra desaturation step, potentially achieved by stearoyl-acyl carrier protein desaturases (SAD). Later, three putative SAD genes (SAD1-SAD3) were isolated [29]. Transgenic expression and in vitro enzyme assays revealed SAD2 to be a functional desaturase capable of introducing 18:1 Δ^9^ and 16:1 Δ^4^ fatty acid intermediates, from which it was hypothesized that (*Z*)-9 alkenes and (*Z*)-12 alkenes are built. Three additional putative SAD genes (SAD4-SAD6) were also identified from an *O. sphegodes* transcriptome [30].

In *O. sphegodes* and *O. exaltata*, SAD1 and SAD2 expression levels were shown to be significantly correlated with (*Z*)-9 and (*Z*)-12-alkene production, while high SAD5 expression was correlated with the (*Z*)-7-alkene production unique to *O. exaltata* [31]. In vitro enzyme activity studies further showed that a putative housekeeping desaturase, SAD3, catalyses the general reactions of stearate to oleate (18:0-ACP to 18:1 Δ^9^-ACP), and palmitate to palmitoleate (16:0-ACP to 16:1 Δ^9^-ACP), whereas SAD5 is a specialized 16:0 Δ^9^-ACP enzyme [32]. Subsequent elongation of a 16:1 Δ^9^-ACP to a 26:1 Δ^19^-coenzyme A precursor, followed by decarbonylation, would yield the (*Z*)-7 alkene (25:1 Δ^7^) that characterizes *O. exaltata*.

In *O. speculum*, the pollinator attractants were also identified as carboxylic acid derivatives [17]. The most attractive compounds from both floral extracts and females of the scoliid wasp pollinator *Campsoscolia ciliata* were (ω-1)-hydroxy- and -oxo acids. However, it is noteworthy that the pseudo-copulation rates in field bioassay experiments more than doubled when aldehydes such as (*Z*)-9-octadecenal and octadecanal, together with the esters ethyl linoleate and ethyl oleate, were added to the dummy female [17].

The phylogenetic relationships within *Ophrys* are currently under debate [33,34,35,36,37], with some phylogenetic analyses indicating that the *Argogorytes*-pollinated *O. insectifera* group represents a basal taxon, while the latest studies place the *O. fusca* complex, including *O. iricolor*, as ancestral [36,37]. All studies agree that wasp pollination is ancestral to bee pollination in *Ophrys*. 

To obtain a broader understanding of the chemical details of semiochemicals in the wasp-pollinated *O. insectifera*, and sex pheromone candidates in its pollinator *A. fargeii*, we used GC-EAD, GC-MS, microderivatisation reactions, and organic synthesis to identify EAD-active compounds. These semiochemicals were compared with previously identified pollinator attractants from the bee-pollinated *O. sphegodes* and wasp-pollinated *O. speculum*, and biosynthetic relationships within *Ophrys* were proposed. 

## 2. Results and Discussion

**Figure 1:**
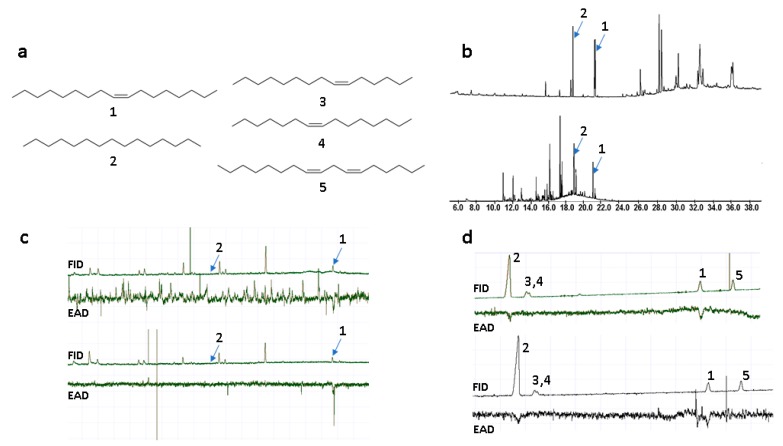
(**a**) Semiochemicals from *Ophrys insectifera* (**1**–**2**; **1** = (*Z*)-8-heptadecene, **2** = *n*-pentadecane) and female *Argogorytes mystaceus* (**1**–**5**; **3** = (*Z*)-6-pentadecene, **4** = (*Z*)-7-pentadecene, **5** = (*Z*,*Z*)-6,9-heptadecadiene). (**b**) GC-MS total ion chromatograms of female *A. fargeii* (upper trace) and *O. insectifera* (lower trace). (**c**) GC-EAD of SPME extracts of *O. insectifera* to antenna of *A. mystaceus* males. Two replicated analyses are shown. (**d**) GC-EAD of synthetic standards **1**–**5** to antenna of *A. mystaceus*. Two replicated analyses are shown.

To identify semiochemicals in *O. insectifera*, and sex pheromone candidates in *Argogorytes fargeii* pollinators, solvent extractions of flowers and insects, and floral headspace sampling, were conducted. Samples of *O. insectifera* labella were extracted in solvents of increasing polarity, from *n*-hexane, to dichloromethane, to methanol. Headspace volatile sampling was performed using solid phase extraction (SPME). Furthermore, whole females of *A. fargeii* were extracted in dichloromethane. Due to the very limited number of pollinators available, we were restricted to evaluating biological activity using gas chromatography coupled with electroantennography (GC-EAD). Since we were unable to locate males of *A. fargeii*, GC-EAD was used to detect which components of the various extracts were detected by *A. mystaceus*, a closely related species that is the second main pollinator of *O. insectifera* [9]. Two compounds from the floral extracts were repeatedly EAD-active (elicited responses in six out of 10, and two out of 10 EAD experiments). These two compounds were tentatively identified by mass spectrometry (GC-MS) as a C17 alkene and *n*-pentadecane. In previous studies on *O. insectifera*, *n*-pentadecane (**2**, Figure 1a) was indeed found to be active in EAG experiments, while no alkenes were isolated or identified [11]. Here, we found that *n*-pentadecane and the C17 alkene were present in the female *A. fargeii* (six extracts of individual insects) and were also present in only minor amounts in floral solvent extracts (three extracts of 10 flowers). We investigated the double bond location by dimethyldisulfide (DMDS) microderivatisation of a semi-preparative GC purified compound that was extracted from the wasp. The observation of identical retention times and mass spectra between the semiochemical isolated from the wasp and the synthesized (*Z*)-8-heptadecene (**1**), before and after treatment with DMDS, meant that the double bond position and configuration of the natural product could be confirmed. Furthermore, a floral extract was treated analogously, and was confirmed to contain identical mass fragments at the same relative intensity and retention time, confirming that the compound detected by *A. mystaceus* was shared between *O. insectifera* and female *A. fargeii.* In addition to the semiochemicals identified from flowers, another two C15-alkenes and one C17-diene were identified from females of *A. fargeii*. These compounds were also isolated by semi-preparative GC and treated with DMDS. Candidate compounds were synthesized and co-injected with natural extracts (on two GC columns) and tested with GC-EAD. The monoenes were subsequently confirmed as (*Z*)-6-pentadecene (**3**) and (*Z*)-7-pentadecene (**4**), while the diene was identified as (*Z*,*Z*)-6,9-heptadecadiene (**5**) (Figure 1).

The GC-EAD and GC-MS analyses of the floral extracts showed that *n*-pentadecane (**2**) was of low abundance and was electrophysiologically active in only two experiments, while (Z)-8-heptadecene (**1**) was active in six experiments. When tested as synthetics at higher concentrations (100 ng to 1 µg), both compounds were strongly EAD-active in replicated experiments. However, the additional alkenes **3**–**5** from *A. fargeii*, when tested as synthetic samples at the higher concentration, elicited consistently less frequent and/or weaker EAD responses compared to the orchid-produced **1** and **2** (Figure 1, Table 1).

By analysing the GC-MS traces of floral extracts, it was observed that larger amounts of compounds **1** and **2** were present in headspace samples of flowers compared with solvent extracts. Although headspace extractions and solvent extractions are not directly comparable, our findings indicate that the flowers likely continuously produce compounds (indicated by increasing quantity with an increase in SPME sampling time), rather than depend on stored compounds (indicated by very low amounts in solvent extracts) in the floral tissue. This observation is in agreement with earlier studies of *O. insectifera* and *O. sphegodes,* favouring headspace sorption extraction over solvent extraction [14,38]. In addition to comparing observations between various *Ophrys* systems, it is of further interest to extend this comparison to other sexually deceptive orchids with known semiochemistry. Such cases are predominantly Australian, where the pollinator attractants in hammer orchids and spider orchids, unlike in *Ophrys*, have been found to be stored in relatively large amounts within the floral tissue [21,23,25].

**Figure 2:**
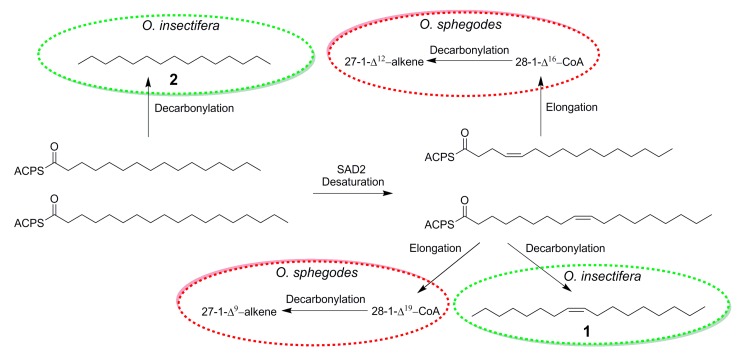
Proposed biosynthesis of bioactive alkenes in *Ophrys sphegodes* (from [32]) and *O. insectifera.*

The discovery of (*Z*)-8-heptadecene (**1**) in *O. insectifera,* detected by males of *A. mystaceus,* provides important insights about the chemistry of *Ophrys* orchids. In earlier studies of the biosynthetic pathways for the longer chained C_25_ and C_27_ alkenes from *O. exaltata a*nd *O. sphegodes*, C_16_- and C_18_ activated carboxylic acids have been proposed as intermediates [32] (Figure 2). In fact, it has been proposed that in the plastid of the lip epidermis cell of the labellum of *O. exaltata* and *O. sphegodes*, 16:0-ACP and 18:0-ACP are transformed to 16:1 Δ^4^-ACP and 18: 1 Δ^9^-ACP by SAD2, before being elongated in the cuticle [29]. If instead, 16:0-ACP and 18:1 Δ^9^-ACP are decarbonylated, the exact compounds found to be EAD-active in *O. insectifera*, *n*-pentadecane (**2**) and (*Z*)-8-heptadecane (**1**), would be formed (Figure 2).

In a similar manner, our results can be compared to the pollinator attractants previously identified in *O. speculum*. Out of the blend of eight electrophysiologically active compounds that showed the highest pollinator attraction in field bioassays, three compounds: hexadecanal, (*Z*)-9-octadecenal, and ethyl oleate, show strong structural similarity with the hydrocarbons that we identified in *O. insectifera*. In fact, decarbonylation of these semiochemicals, in a similar way as proposed in the case of *O. sphegodes* (Figure 2), would yield pentadecane (**2**) from hexadecanal and (*Z*)-8-heptadecene (**1**) from (*Z*)-9-octadecenal and ethyl oleate.

Compared to the recent studies of Australian *Drakaea* and *Caladenia* orchids, where multiple, structurally diverse pollinator attractants have been identified in multiple species [21,22,23,25], the structural similarities between the semiochemicals of *O. insectifera*, *O. sphegodes*, and *O. speculum* are evident, all being clearly biosynthetically closely related carboxylic acid derived compounds. It is also interesting to note the difference in volatility compared to the widely studied Australian systems, where “traditional” volatiles are used as long-range attractants, while the European systems utilise less volatile cuticular hydrocarbons, such as the C_27_–C_29_ alkenes in *O. sphegodes,* which have been proven sufficiently volatile to lure pollinators from a distance as attractants [15]. Furthermore, it is relevant to note that in the case of *O. insectifera* and *A. fargeii*, the orchid and pollinator share the exact same semiochemicals, which is in agreement with other investigated *Ophrys* systems, including *O. sphegodes* [39] and *O. speculum* [17], as well as with most Australian systems [4] (but see [40]).

In conclusion, we have identified (*Z*)-8-heptadecene (**1**) and pentadecane (**2**) as shared semiochemicals from *O. insectifera* and *A. fargeii*. Access to denser populations of *A. fargeii* or *A. mystaceus* would be required to undertake bioassays testing the field activity of these compounds as pollinator attractants. Nevertheless, this study provides an important first step in the identification of key compounds that, once pollinator populations have been located, are available to be tested in field behavioural bioassays. Furthermore, the identification of these semiochemicals and comparison with related species within the genus shows strong commonalities in structures and suggests a conserved biosynthetic pathway for semiochemical production within *Ophrys*.

## 3. Materials and Methods

### 3.1. General Experimental Procedures

NMR spectra (Supplementary Materials) were acquired on a Bruker Avance (Bruker, Billerica, MA, USA) 500 or 600 MHz spectrometer with CDCl_3_ as solvent. Chemical shifts were calibrated to resonances attributed to the residual solvent signal.

EIMS (70 eV) were recorded on an Agilent 5973 mass detector connected to an Agilent 6890 GC equipped with a DB5-MS column (Agilent, Santa Clara, CA, USA, 50 m × 0.2 mm × 0.33 µm) using helium as the carrier gas.

Semi-preparative gas chromatography was performed on an HP 5890 GC (Agilent, USA), equipped with a three-way glass splitter separating the gas flow post column into the FID and the collector. An RTX-5 column, 30 m × 0.53 mm id × 5 µm film (Restek, Bellefonte, PA, USA) was used. Samples of 3 µL were injected in splitless mode (1 min) and helium was used as the carrier gas. A custom-made manual fraction collector was used, with samples collected in glass capillaries (100 × 1.55 mm id, Hirschmann Laborgeräte, Eberstadt, Germany) positioned in an aluminium holder submerged in a dry ice/acetone bath. All fractions were eluted with dichloromethane and stored at −20 °C until used for microderivatisation experiments [26].

GC-EAD data were recorded using an Agilent 6890 GC equipped with an identical column as the GC-MS and a flame ionization detector (FID) using helium as carrier gas. A GC effluent splitter (split ratio 1:1) was used to split the flow to the FID and EAD. The split for EAD was passed through a Syntech effluent conditioner (Syntech, Buchenbach, Germany) containing a heated transfer line, with the outlet placed in a purified and humidified airstream, where the electrodes holding the antenna were presented. For each EAD run, an excised antenna with the tip cut off, was mounted on a holder consisting of two electrodes using electrode gel. The electrode was connected to a PC via a serial Syntech intelligent data acquisition controller (IDAC) interface for simultaneous recording of the FID and EAD signals in the Syntech software package.

Solvents for extractions and purifications were of HPLC grade.

### 3.2. Plant Material and Insects

All plants and insects were collected in June over four years (2016–2019) at various field locations in Sweden. *Ophrys insectifera* were sourced from several populations across the central parts of Öland. Flowers were kept in cooler boxes (ca. 4 °C) while transported to the laboratory, where they were either sampled with solid-phase microextraction (SPME), extracted with hexane, dichloromethane or methanol, or kept as baiting flowers to collect male insects. Male *Argogorytes mystaceus* were collected from *O. insectifera* flowers on stems (20 flowers) near Torslunda, Öland or near Södertälje, Södermanland. Female *Argogorytes fargeii* were collected from food plants, *Pastinaca sativa*, near Långöre, Öland.

### 3.3. Extraction and Isolation

Flowers for SPME were enclosed in oven bags (Multix 25 cm × 38 cm, McPherson’s Limited, Kingsgrove, NSW, Australia) and sampled for 24 h (DVB/CAR/PDMS, Supelco, Bellefonte, PA, USA) at room temperature. For solvent extractions, labella were removed and batches of 20 were extracted in each solvent (ca. 1 mL) for 48 h. The extracts were concentrated under a gentle stream of nitrogen at room temperature to a final volume of ca. 0.1 mL, stored at −20 °C and subsequently used for GC-MS and GC-EAD analyses. Female *A. fargeii* were extracted in dichloromethane (ca. 0.5 mL) for 48 h. The extracts were concentrated and treated as for the floral extracts.

### 3.4. Structure Elucidations of Alkenes

From a concentrated dichloromethane extract of a female *A. fargeii,* the two fractions containing C15-alkenes and C17-alkenes were isolated by semi-preparative GC. Each fraction, in hexane (30 µL), was treated with DMDS (50 µL) and iodine in diethyl ether (5 µL, 60 mg/mL). The reaction mixtures were left at 40 °C in vials over night before being washed with sodium bisulphite (5%) and concentrated to ca. 20 µL under nitrogen before being analysed by GC-MS [41]. The fraction containing C15-alkenes contained two compounds, with characteristic ions for 6-pentadecene (M = 304, fragments *m*/*z* = 131, 173) and 7-pentadecene (M = 304, fragments *m*/*z* = 145, 159). The fraction containing C17-alkenes contained one monoene and one diene, with characteristic ions for 8-heptadecene (M = 332, fragments *m*/*z* = 159, 173, also present in *O. insectifera*) and 6,9-heptadecene (M = 362, fragments *m*/*z* = 131, **155**, 159, **183**, 203 and 231) [42].

### 3.5. Preparation of Alkenes

All alkenes apart from 7-pentadecene were prepared from the corresponding C16- and C18-carboxylic acids (oleic acid, linoleic acid, palmitoleic acid) via a modified Barton reductive decarboxylation [43]. 7-Pentadecene was synthesised via 7-pentadecyne, prepared from 1-bromoheptyne and octylmagnesium chloride [44]. The alkyne was partially reduced to the *cis*-alkene in a low yield by the method of Obora et al. [45], although in amounts sufficient to our needs.

## Figures and Tables

**Table 1:** Occurrence of semiochemicals in *Ophrys insectifera* (SPME extracts) and *Argogorytes fargeii* females (solvent extracts), with electroantennographic responses in *A. mystaceus* males.

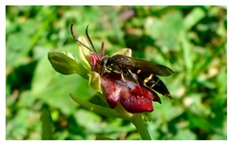 *Argogorytes mystaceus* visiting *Ophrys insectifera*	**Compound**	**Abundance in** ***O. insectifera***	**Abundance in** ***A. fargeii* (Female)**	**EAD-Activity**
	**1**	✔✔	✔✔	✔✔
	**2**	✔	✔✔	✔
	**3**	–	✔	(✔)
	**4**	–	✔	(✔)
	**5**	–	✔✔	(✔)

✔✔= very abundant compound (>20% of base peak area); repeated (6 extracts, >6 synthetic samples) strong EAD-responses. ✔ = abundant compound (>10% of base peak area); repeated EAD-responses (2 extracts, >6 synthetic samples). (✔) = occasional weaker EAD-response (generally less than 50% of response of orchid semiochemicals, >3 synthetic samples). Photo A.M. Weinstein.

## Supplementary Materials

Supplementary materials (NMR spectra for all semiochemicals) can be found at https://www.mdpi.com/1422-0067/21/2/620/s1.

## Abbreviations

EAG	Electroantennography
GC	Gas chromatography
EAD	Electroantennographic detection
SAD	Stearoyl-acyl carrier protein desaturase
ACP	Acyl-carrier protein
EIMS	Electron impact mass spectrometry
GCMS	Gas chromatography mass spectrometry
NMR	Nuclear magnetic resonance
FID	Flame ionization detector
HPLC	High-pressure liquid chromatography
SPME	Solid-phase microextraction
DVB	Divinyl benzene
CAR	Carboxen
PDMS	Polydimethylsiloxane
DMDS	Dimethyl disulphide
