# Supplementary material for: Identification of (Z)-8-Heptadecene and n-Pentadecane as Electrophysiologically Active Compounds in Ophrys insectifera and Its Argogorytes Pollinator
Source: Int J Mol Sci. 2020 Jan 17;21(2):620. doi: 10.3390/ijms21020620 (PMC7014428; doi:10.3390/ijms21020620)

Identification of (*Z*)-8-heptadecene and *n*-pentadecane as electrophysiologically active compounds in *Ophrys insectifera* and its *Argogorytes* pollinator

Björn Bohman, Alyssa M. Weinstein, Raimondas Mozuraitis, Gavin R. Flematti and Anna-Karin Borg-Karlson

Supplementary Materials

NMR-spectra of synthetically prepared compounds **1**, **3**, **4** and **5**

^{1}H NMR (600 MHz, $CDCl_3$)

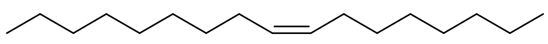

1

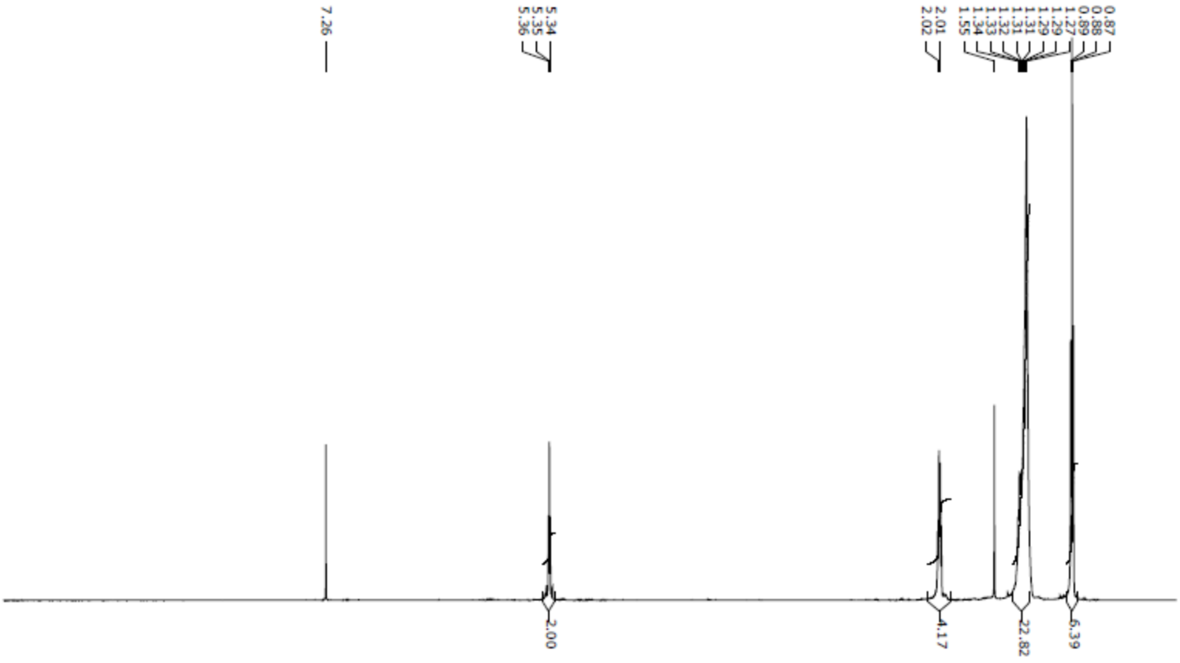

^{13}C NMR (150 MHz, $CDCl_3$)

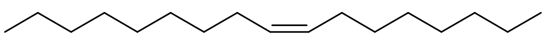

1

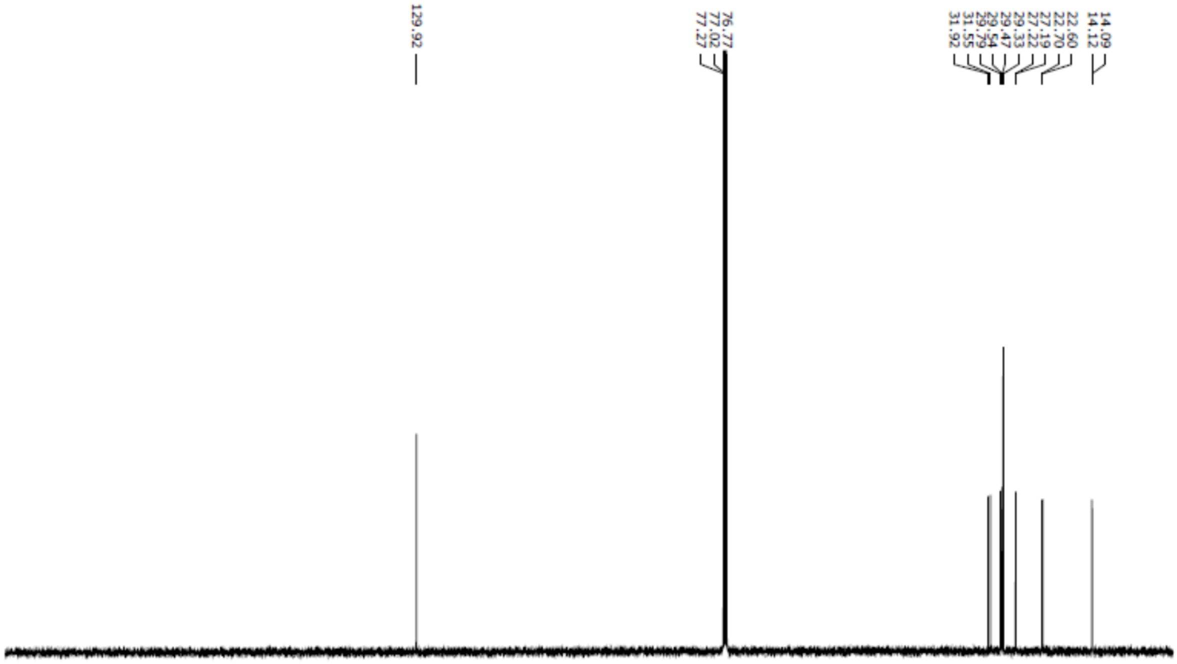

^{1}H NMR (600 MHz, $CDCl_3$)

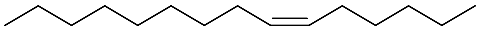

3

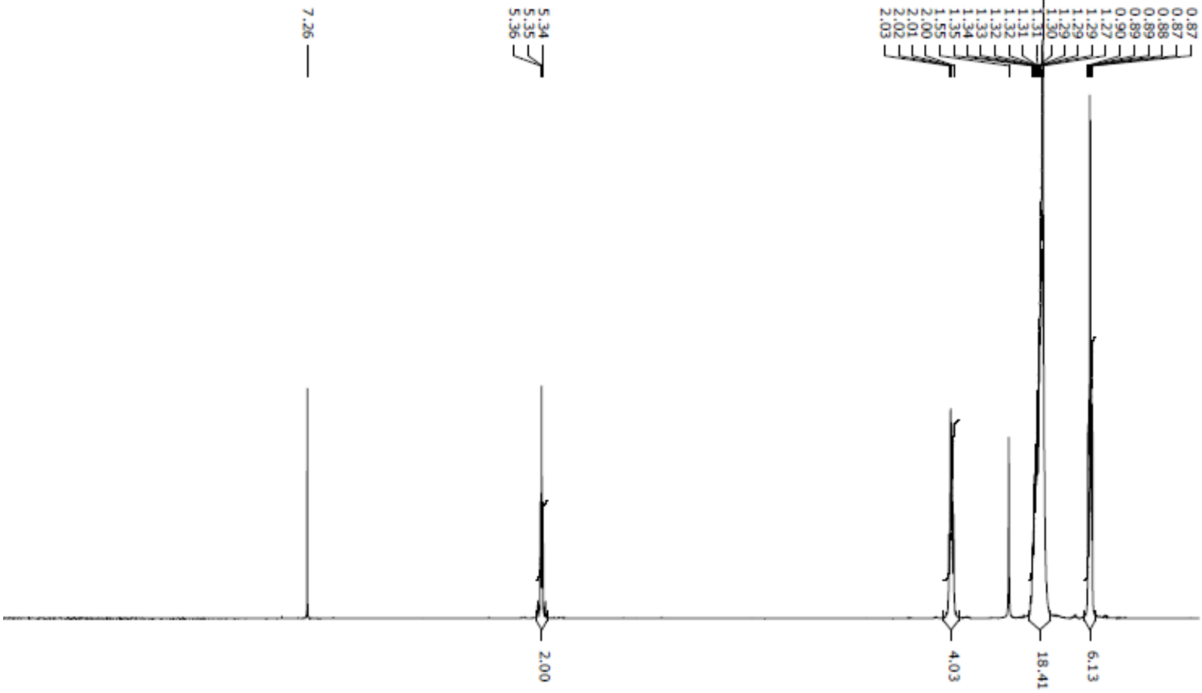

^{13}C NMR (150 MHz, $CDCl_3$)

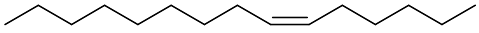

3

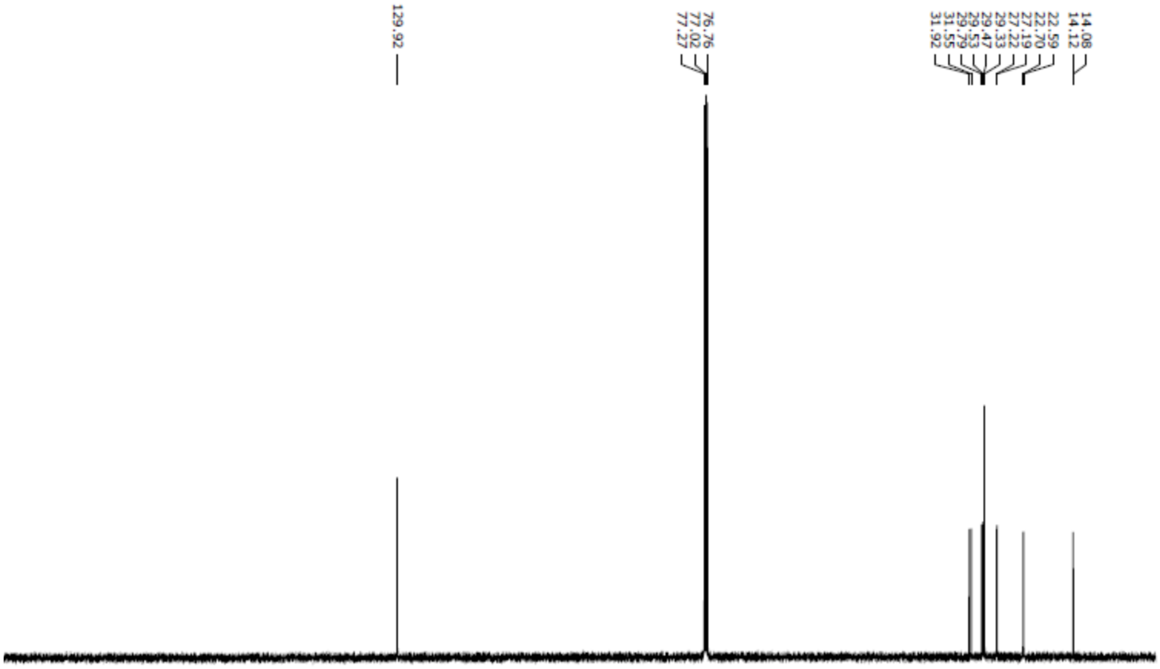

^{1}H NMR (600 MHz, $CDCl_3$)

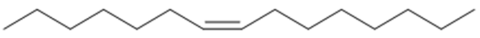

4

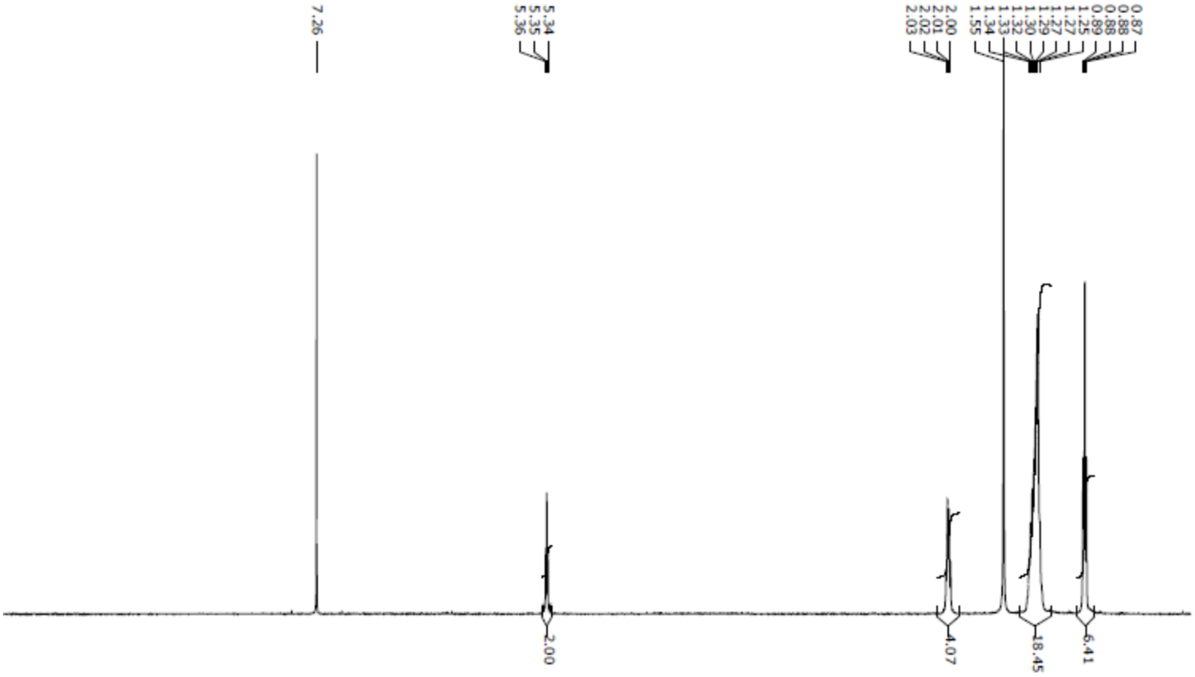

^{13}C NMR (150 MHz, $CDCl_3$)

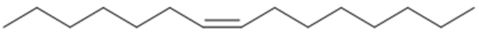

4

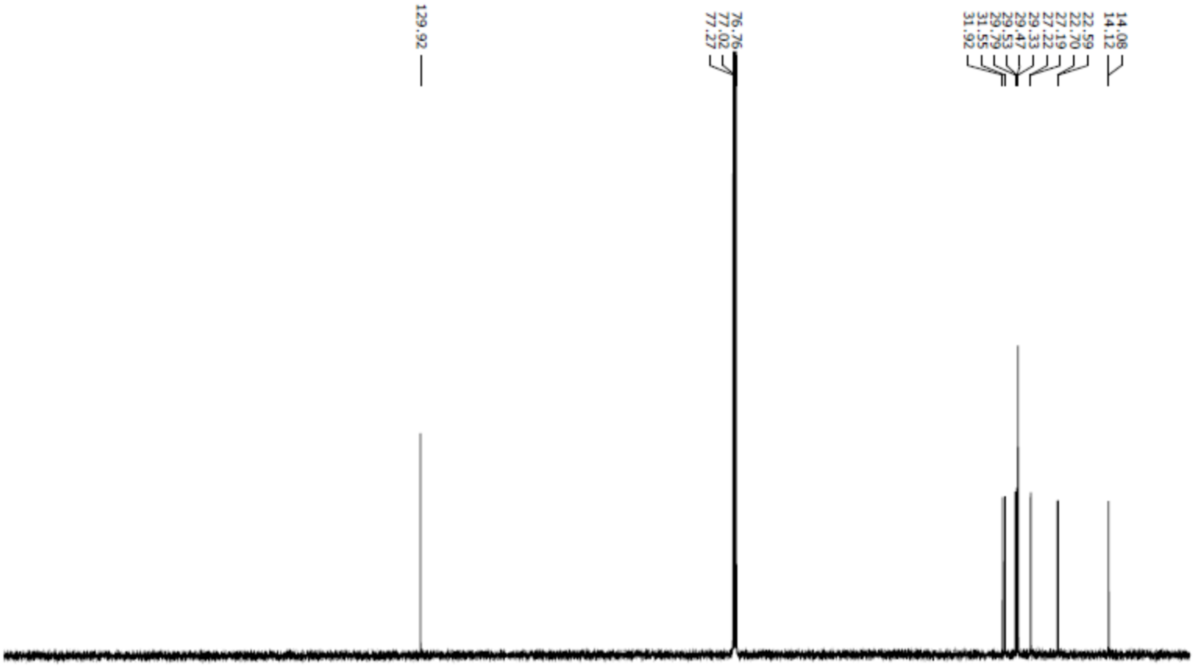

^{1}H NMR (500 MHz, $CDCl_3$)

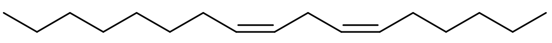

5

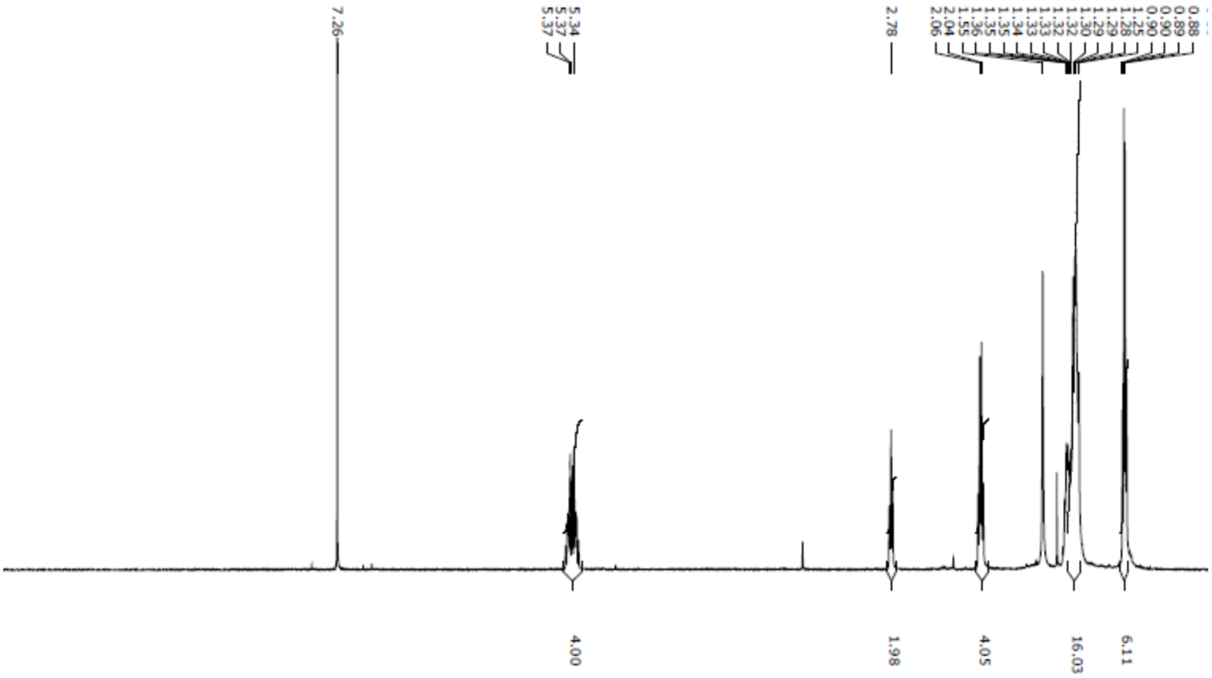

^{13}C NMR (125 MHz, $CDCl_3$)

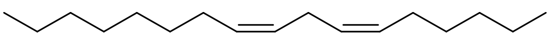

5

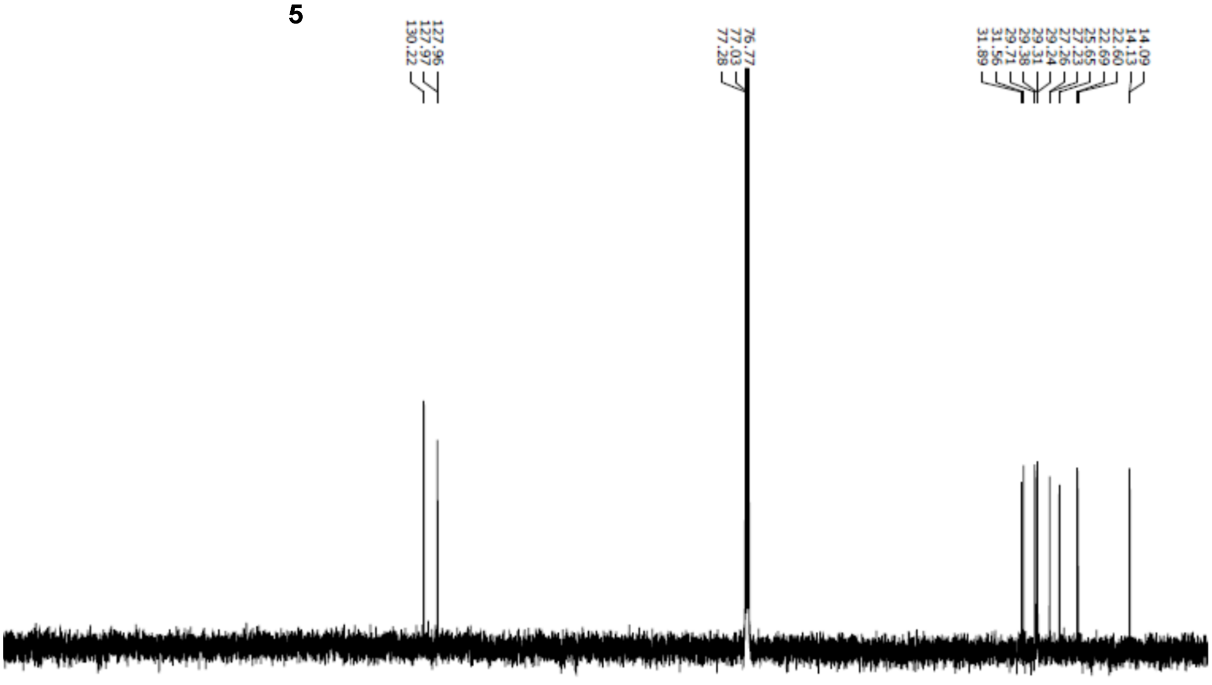

Supplement: Supplementary file 1 [file ijms-21-00620-s001.pdf]
